# Hepatitis E Virus Genotype Diversity in Eastern China

**DOI:** 10.3201/eid1610.100873

**Published:** 2010-10

**Authors:** Wen Zhang, Yilin He, Hua Wang, Quan Shen, Li Cui, Xiaochun Wang, Shihe Shao, Xiuguo Hua

**Affiliations:** Author affiliations: Jiangsu University, Jiangsu, People’s Republic of China (W. Zhang, H. Wang, X. Wang, S. Shao);; Taizhou Center for Disease Control and Prevention, Jiangsu (Y. He);; Ohio State University, Wooster, Ohio, USA (Q. Shen);; Shanghai JiaoTong University, Shanghai, People’s Republic of China (L. Cui, X. Hua)

**Keywords:** Hepatitis E virus, viruses, genotype diversity, phylogenetic analysis, zoonoses, swine, China, dispatch

## Abstract

We studied 47 hepatitis E virus (HEV) isolates from hospitalized patients in Nanjing and Taizhou, eastern China. Genotypes 1, 3, and 4 were prevalent; genotype 3 and subgenotype 4b showed a close relationship with the swine strains in eastern China, thus indicating that HEV genotype 3 had infected humans in China.

*Hepatitis E virus* (HEV), genus *Hepevirus*, is a nonenveloped virus with a positive-stranded RNA genome of ≈7.2 kb (*1*). Researchers have hypothesized that zoonotic infection is involved in HEV transmission (*2**,**3*). HEV isolates have been divided into 4 distinct genotypes, and further classification of the 4 genotypes into 24 subtypes has been proposed (*4*). Genotypes 1 and 2 have been identified exclusively in humans, and genotypes 3 and 4 have been found in humans and several animal species. Genotypes 1 and 2 have been isolated in Asia, Africa, and North America; genotype 4 has been identified only in Asia; and genotype 3 has been found in almost every country**.** Although genotype 3 was found to be prevalent in swine populations in China in 2007 (*5*), this viral genotype has not previously been reported in humans in this country**.** Our study aimed to characterize the genotype diversity of the strains and to determine the full-length sequence of the most prevalent genotype in eastern China.

## The Study

We studied 47 HEV isolates from patients (15 women and 32 men, 19–73 years of age) admitted to hospitals in Nanjing (26 patients) and Taizhou (21 patients) in eastern China and who were serologically confirmed to have HEV infection by commercial ELISAs (Wan Tai Pharmaceutical, Beijing, People’s Republic of China). HEV RNA was detected by reverse transcription–PCR as described (*6*). A serum sample negative for HEV was included as a control for testing sample contamination.

**Figure 1:**
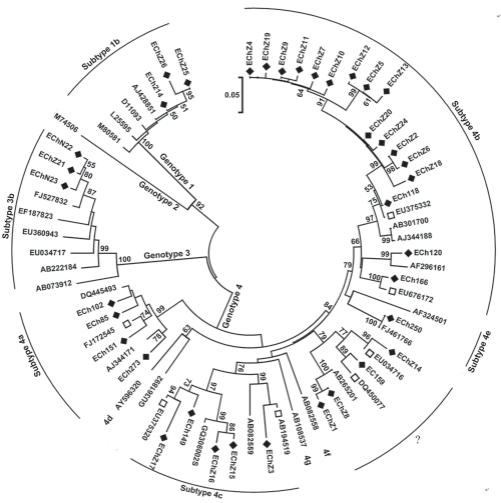
Phylogenetic tree of hepatitis E virus isolates in eastern China, 2007–2009. The phylogenetic tree was produced with a 348-nt open reading frame 2 sequence alignment of 37 isolates from this study and other 31 reference sequences, using the neighbor-joining method and evaluated by using the interior branch test method with MEGA4 software (www.megasoftware.net). Percentage of bootstrap support is shown by values at the branch nodes of the tree. Only nodes with a bootstrap value >50 are labeled; these values are the result of resampling the data 1,000 times. Black diamonds, isolates identified in the current study; white squares, GenBank sequences with the highest sequence homology to our sequences; ?, genotype 4 strains that could not be subtyped but closely clustered with each other, forming a new subtype. Scale bar indicates nucleotide substitutions per position.

Sequence analysis based on the PCR-amplified products (primer sequences were removed) indicated that among the 47 HEV strains, 31 belonged to genotype 4, 13 belonged to genotype 1, and 3 belonged to genotype 3, which indicated that genotype 4 is the main type prevalent in humans in eastern China. The 13 genotype 1 isolates shared >98% sequence homology and showed 3 distinct nucleotide sequences. Figure 1 shows the phylogenetic tree constructed for the 37 distinct sequences in this study and their closest matching sequences in GenBank. To further classify the subgenotype of these strains, we included in the phylogenetic analysis some well-characterized subtype strains (*4*).

Results indicated that the 31 genotype 4 isolates found in this study were divided into 5 different subtypes. Seventeen of the genotype 4 isolates were subtyped as 4b, according to the reference sequences (GenBank accession nos. AJ344188 and AB116161), and they shared 93.2%–99.7% sequence homology among themselves. Strains ECh250 shared 100% sequence homology with FJ461766 and could be classified into subtype 4e, according to the reference sequence (AF234501). Five of the isolates, which shared 89.4%–91.2% sequence identities, belonged to subtype 4d, defined by reference strain AB082559. Four of the strains shared 92.4%–95.7% sequence homology and could be defined as subtype 4a, according to the reference stsrain (AJ344171).

Notably, the other 4 genotype 4 isolates could not be subtyped on the basis of the reference strains described by Lu et al. (*4*) but closely clustered with each other, forming a new subtype (Figure 1). The 3 genotype 1 isolates identified in this study shared >98% sequence homology and could be defined as subtype 1b, a virus subtype mainly prevalent in China (*4*). The 3 genotype 3 isolates in this study shared 97.7%–98.6% sequence identities and were closely related (96.4%–97.9% sequence identities) to a swine isolate (FJ527832), found in swine in the Shanghai area (*7*).

Genotype 4 HEV has been found to be the dominant cause of hepatitis E in China and has been involved in zoonotic transmission in eastern and southern China (*3**,**8*). When a BLAST (http://blast.ncbi.nlm.nih.gov/Blast.cgi) search in GenBank was performed, we found that some genotype 4 isolates obtained in this study shared the highest sequence homology with previous swine strains (Figure 1). For subtypes 4a, 4c, and 4e and the new subtype groups, the swine strains that showed the closest relationship with our studied strains had been isolated from swine in other regions of China; this finding suggests that these isolates might not be indigenous to eastern China (Figure 1). Fifteen of the genotype 4 isolates in the 4b group had >96.3% sequence identity and closely clustered with a swine strain (EU375332) that had been isolated from swine in eastern China (*9*); strain ECh118 had 99.3% sequence homology to this swine strain (Figure 1).

Because the serum specimen from which ECh118 was identified was insufficient for complete genome amplification, EChZ20 was selected for complete genome sequencing using the primers described previously (*10*). The complete genome comprises 7,228 nt, excluding the 3′ poly(A) tail. The open reading frame 1 (ORF1) begins at nt 26 and ends at nt 5140 (5,115 nt); ORF2 (nt 5137–7161) comprises 2,022 nt and encodes 674 aa; ORF3 (nt 5165–5509) comprises 345 nt and encodes 114 aa.

**Figure 2:**
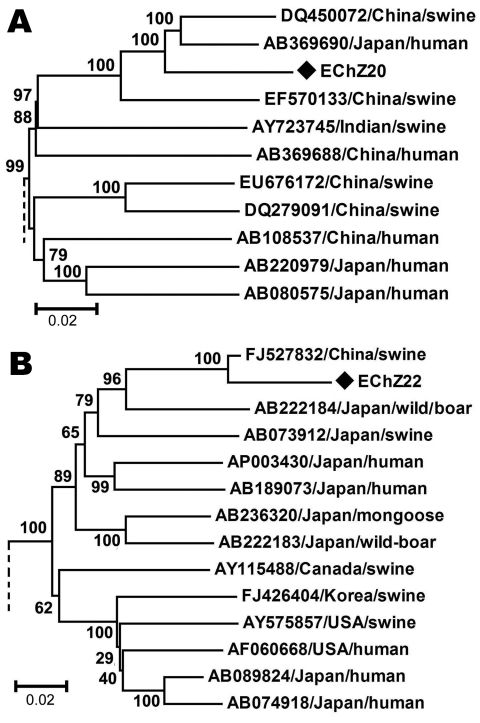
Phylogenetic tree showing alignment of the complete genome of EChZ20 of hepatitis E virus determined in the present study and the referenced genotype 4 isolates with complete genome available in GenBank (A), and the 1,681-nt partial open reading frame 2 sequence of EChN22 and referenced genotype 3 isolates with complete genome available in GenBank (B). The tree was constructed by using the neighbor-joining method and evaluated by using the interior branch test method with MEGA4 software (www.megasoftware.net). Percentage of bootstrap support is indicated at each node. GenBank accession number, source, and country of origin are indicated. Strains identified in this study are indicated by black diamonds. Only partial branches that were sufficient for elucidating the relationship between the study strains and their related strains are shown. Scale bars indicate nucleotide substitutions per site.

The phylogenetic tree obtained for the complete genome of EChZ20 and other 16 representative genotype 4 isolates indicated that EChZ20 closely clustered with DQ450072, EF570133, and AB369690 and shared 90.7%, 91.7%, and 89.1% sequence identities with them, respectively (Figure 2, panel A). DQ450072 and EF570133 were isolated from swine in eastern China (*10*), and AB369690 was isolated from a patient from Japan who had traveled to Shanghai, China, before the onset of acute hepatitis E, according to information in GenBank. These results suggested that subtype 4b virus isolates were involved in cross-species transmission from swine to humans in eastern China.

The genotype 3 isolates found in many countries, whether from swine or humans, have been reported to show a strong genetic relationship (*11**–**13*). Since 2007, genotype 3 has been found in swine groups in several areas in China (*14*); however, no reports have indicated that genotype 3 HEV infects humans in this country. In our study, genotype 3 was detected in humans in eastern China, and the strains found in humans showed high sequence homology (96.4%–97.9%) to the virus strains prevalent in swine in China. We intend to amplify the complete genome of this virus strain; however, because the quantity of serum in this study was limited, only a 1,681-nt partial ORF2 sequence of EChN22 was obtained.

Phylogenetic analysis, based on the 1681-nt sequence of EChN22 and other genotype 3 strains available in GenBank, confirmed that EChN22 belonged to genotype 3b and that it clustered closely with a swine isolate from China (FJ527832) and shared 97.2% nt and 99.6% aa sequence homology with it (Figure 2, panel B). These results suggested that the genotype 3 virus strain prevalent in humans in eastern China might come from swine in this area. The sequences were deposited in GenBank under accession nos. HM439249–HM439285

## Conclusions

Our study indicated that 3 genotypes of HEV (1, 3, and 4) are prevalent in humans in eastern China. The 31 genotype 4 strains could be further divided into 5 different subtypes, including a new subtype that, to our knowledge, has not been subtyped in previous studies. The complete genome of 1 representative strain of the most prevalent subtype (genotype 4b) was sequenced, and it showed a close relationship with swine strains prevalent in swine in eastern China.

Three genotype 3 HEV strains were identified, and one of them was selected and sequenced to obtain a longer sequence (1,681 nt). Phylogenetic analysis based on the 1,681-nt partial ORF2 sequence indicated the genotype 3 strain showed relatively high sequence homology (97.2%) to virus strains recently isolated from swine in eastern China. This finding suggests that the genotype 3 HEV strains prevalent in humans and swine in this area might come from a common infection source.

In the 1980s and early 1990s, genotype 1 was considered the predominant genotype in China; since 2000, genotype 4 HEV has become the dominant cause of hepatitis E disease in China (*15*). Our current study showed that genotype 3 HEV could be associated with human HEV infection in China. These results may provide a hint that China is transitioning from experiencing HEV infection that was primarily associated with HEV strains transmitted along the human-to-human route to strains transmitted zoonotically.
